# Supplementary material for: Super-enhancer-associated gene CAPG promotes AML progression
Source: Commun Biol. 2023 Jun 9;6:622. doi: 10.1038/s42003-023-04973-1 (PMC10256737; doi:10.1038/s42003-023-04973-1)
Supplement: Supplementary file 2 — Supplementary information [file 42003_2023_4973_MOESM2_ESM.pdf]

Supplementary Information:

Supplementary Figure 1

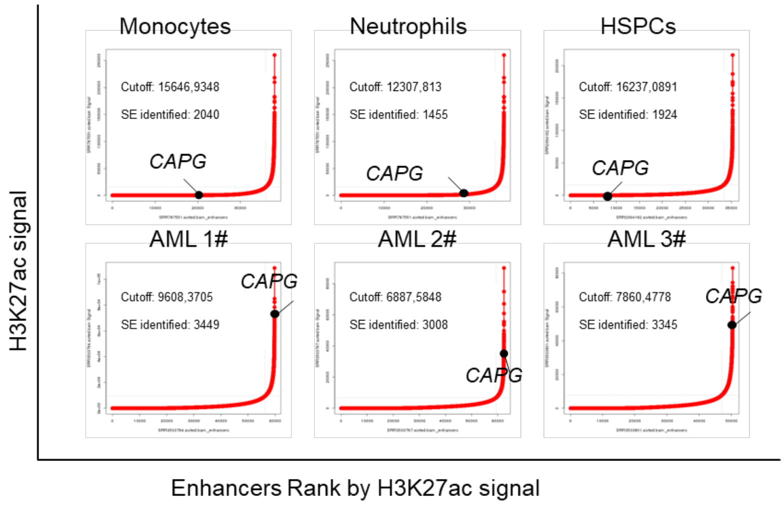

Supplementary Figure1:

Enhancers in three normal blood cells and three AML cells ranked based on H3K27ac signal intensity.

Supplementary Figure 2

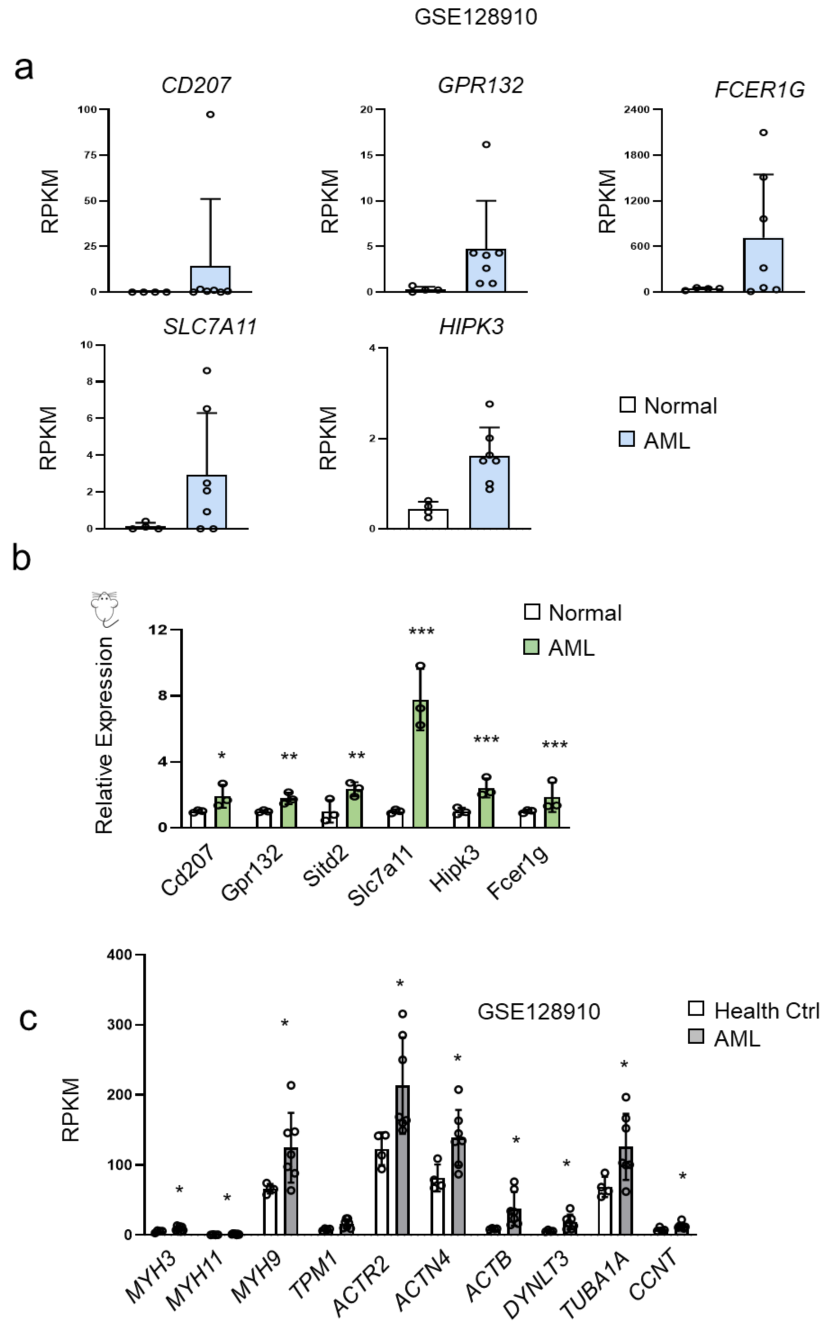

Supplementary Figure2:

a: RNA-seq data (GSE128910）shows AML specific SEs-associated genes are highly expressed in AML patients. Healthy volunteers (n = 4) or AML patients (n = 7).

b: AML-specific SEs-associated genes are highly expressed in MLL-AF9-induced AML mice model.

c: RNA-seq data (GSE128910）shows an increase in the expression of cytoskeletal proteins in AML patients.

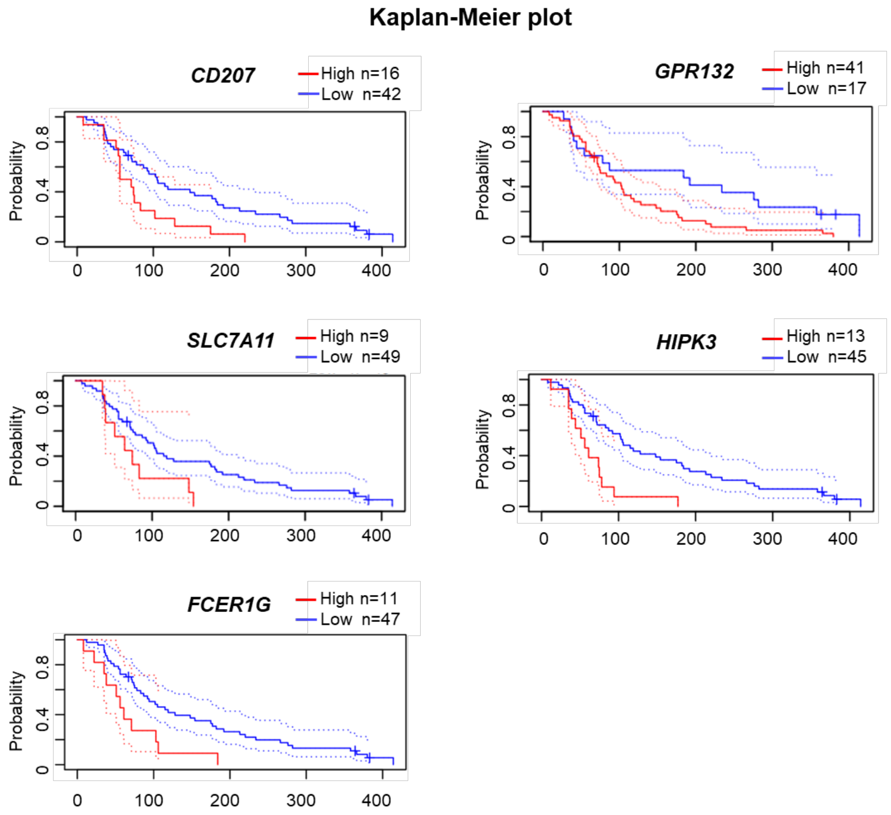

Supplementary Figure 3

The Kaplan–Meier survival curves of AML-specific SE-associated genes in The Cancer Genome Atlas (TCGA)-LAML database.

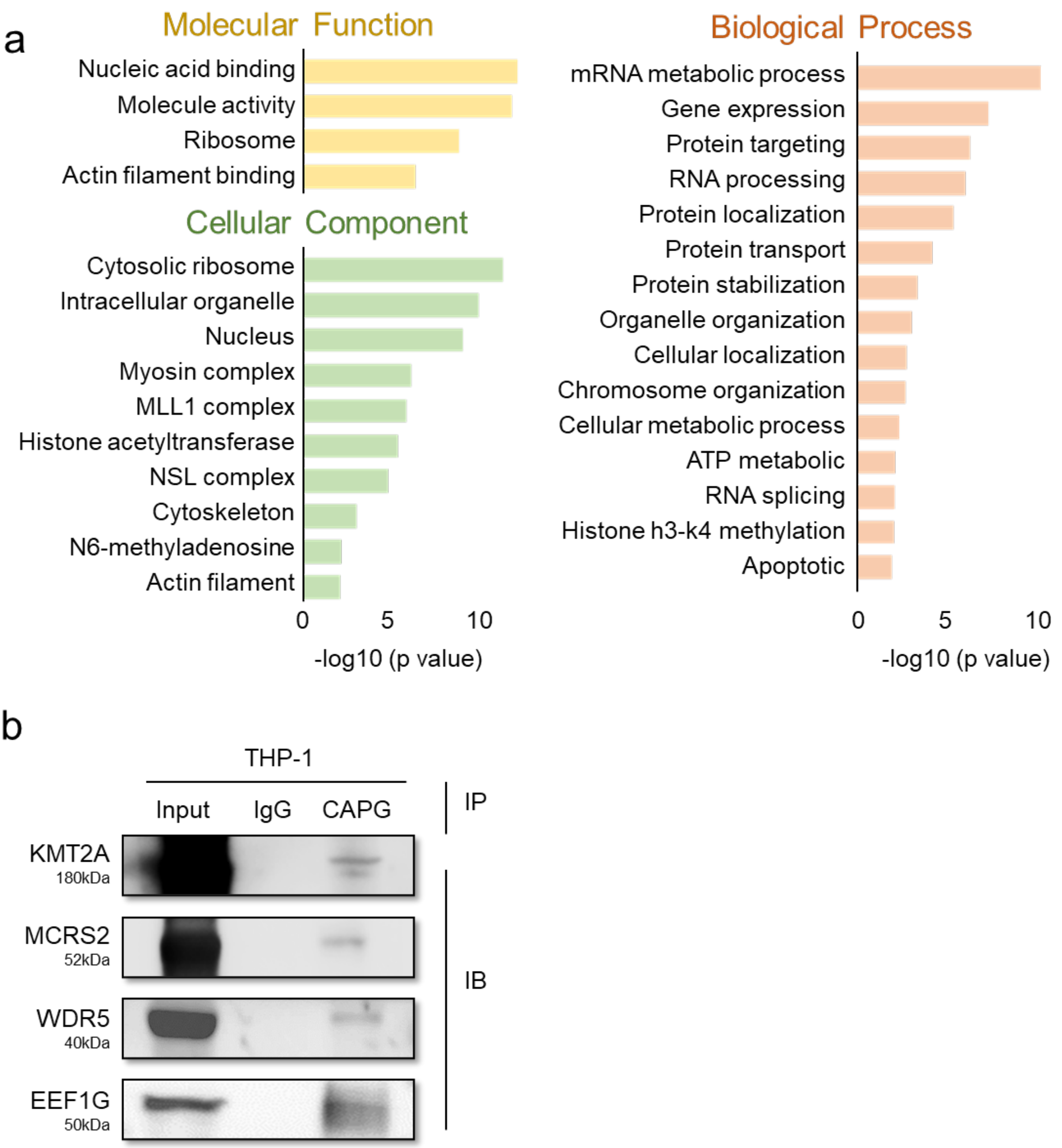

Supplementary Figure 4:

a: Gene ontology (GO) analysis for 79 CAPG interacting proteins. Displayed on the x axis are enrichment scores as calculated by –log 10 (p value).

b: Co-IP shows the interactions between CAPG and NSL complex (WDR5, MCRS1), SNW1 complex (EEF1G), MLL-WDR5 complex (KMT2A, WDR5).

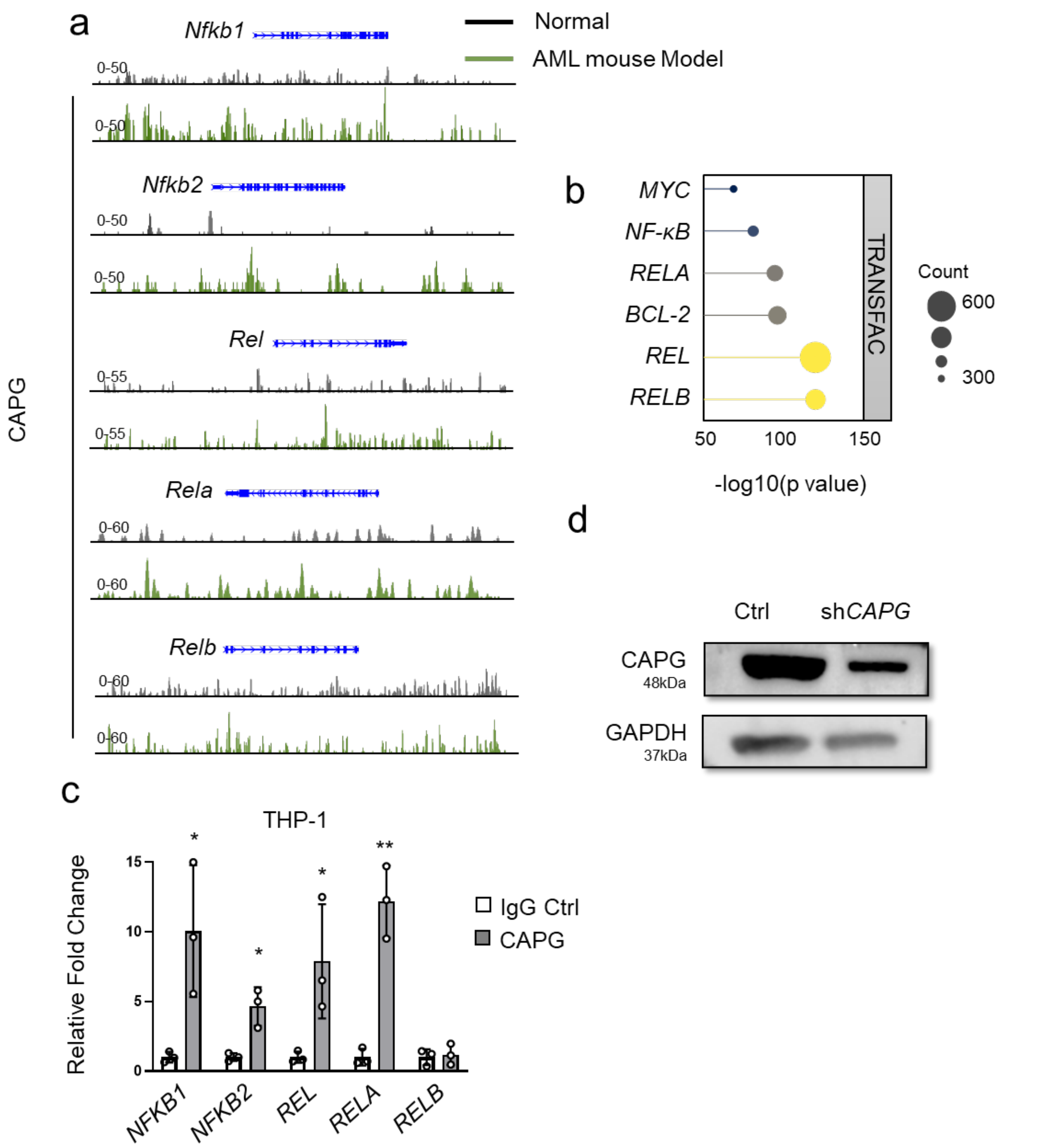

Supplementary Figure 5:

a: Genome browser views of the distribution of *CAPG* ChIP-seq peaks on NF-κB related genes loci.
b: *CAPG* down-stream genes were identified among the predicted targets using TRANSFAC databases in AML cell, P <0.05 for each result displayed on the figure.
c: The binding fold change of *CAPG* at the NF-κB pathway transcription factor family by ChIP-qPCR. Data are presented as means ± SD. *p < 0.05, **p < 0.01. Three biological replicates are assayed for ChIP-qPCR experiment.
d: Western blot analysis showing *CAPG* knockdown in THP-1 cells.

Supplementary Figure 6

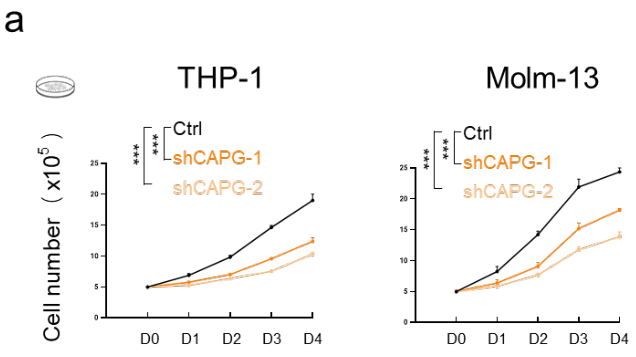

Supplementary Figure 6:

a: In human AML cell lines(THP-1, Molm-13)， cell number in the *CAPG* KD group compared with control.

Supplementary Figure 7

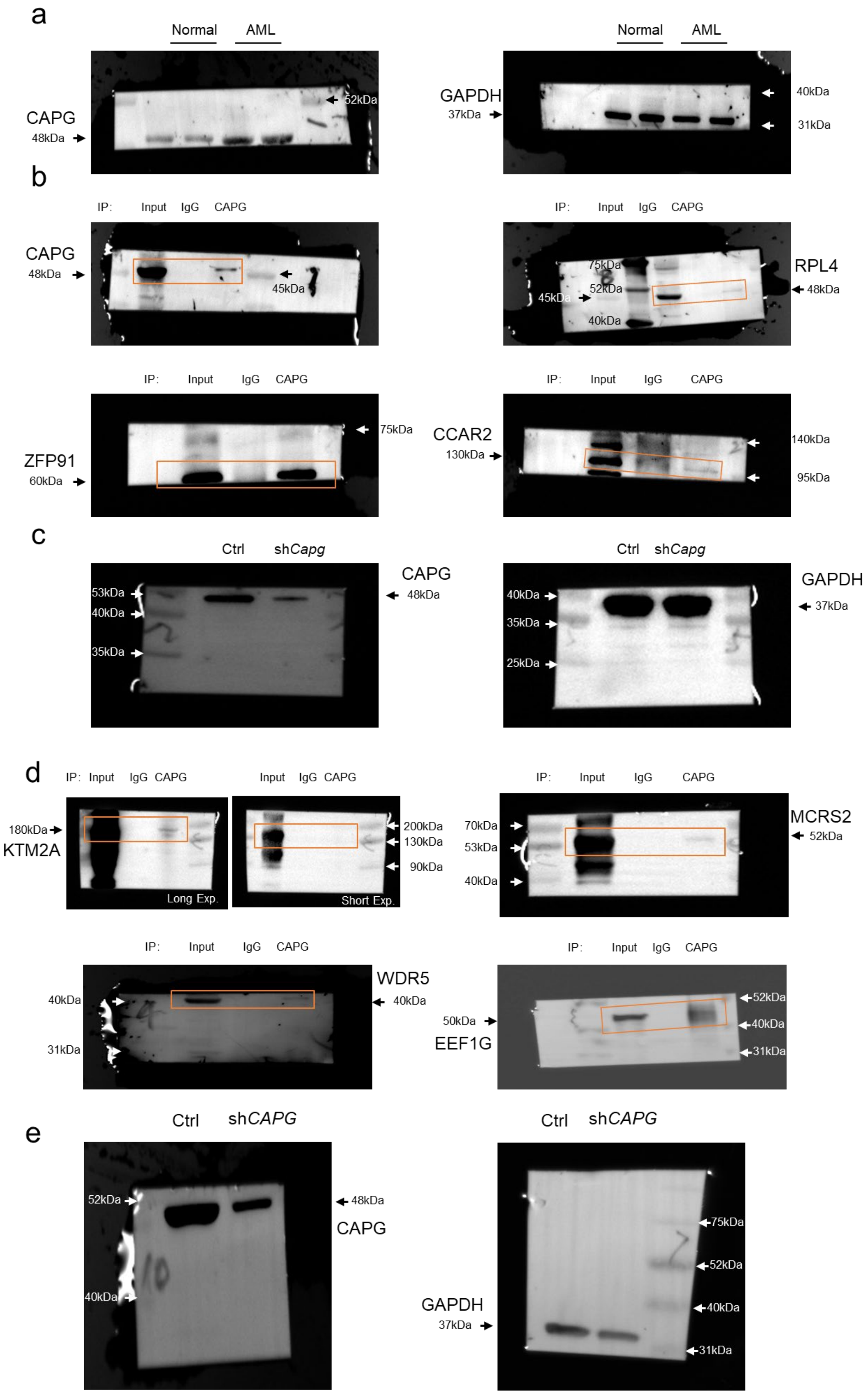

Supplementary Figure 7:

Uncropped western blot images for Figure 2f (a), 3c (b), 5b (c), Supplement Figure 4b (d), 5d (e). This experiment was performed three times, with similar results.
