# Supplementary material for: Super-enhancer-associated gene CAPG promotes AML progression
Source: Commun Biol. 2023 Jun 9;6:622. doi: 10.1038/s42003-023-04973-1 (PMC10256737; doi:10.1038/s42003-023-04973-1)
Supplement: Supplementary file 3 — Description of Additional Supplementary Files [file 42003_2023_4973_MOESM3_ESM.pdf]

Description of Additional Supplementary Files

File name: Supplementary Data 1

Description: CAPG KD RNA-seq data.

File name: Supplementary Data 2

Description: The Oligo information.

File name: Supplementary Data 3

Description: IP-MS resluts.

File name: Supplementary Data 4

Description: Antibody information.
