# Supplementary material for: Super-enhancer-associated gene CAPG promotes AML progression
Source: Commun Biol. 2023 Jun 9;6:622. doi: 10.1038/s42003-023-04973-1 (PMC10256737; doi:10.1038/s42003-023-04973-1)
Supplement: Supplementary file 8 — Reporting Summary [file 42003_2023_4973_MOESM8_ESM.pdf]

Corresponding author(s): Haixia Li
Last updated by author(s): May 2, 2023

Reporting Summary

Nature Portfolio wishes to improve the reproducibility of the work that we publish. This form provides structure for consistency and transparency in reporting. For further information on Nature Portfolio policies, see our Editorial Policies and the Editorial Policy Checklist.

Statistics

For all statistical analyses, confirm that the following items are present in the figure legend, table legend, main text, or Methods section.

n/a	Confirmed	
☐	☒	The exact sample size (n) for each experimental group/condition, given as a discrete number and unit of measurement
☐	☒	A statement on whether measurements were taken from distinct samples or whether the same sample was measured repeatedly
☐	☒	The statistical test(s) used AND whether they are one- or two-sided<br>*Only common tests should be described solely by name; describe more complex techniques in the Methods section.*
☒	☐	A description of all covariates tested
☐	☒	A description of any assumptions or corrections, such as tests of normality and adjustment for multiple comparisons
☐	☒	A full description of the statistical parameters including central tendency (e.g. means) or other basic estimates (e.g. regression coefficient) AND variation (e.g. standard deviation) or associated estimates of uncertainty (e.g. confidence intervals)
☐	☒	For null hypothesis testing, the test statistic (e.g. F, t, r) with confidence intervals, effect sizes, degrees of freedom and P value noted<br>*Give P values as exact values whenever suitable.*
☒	☐	For Bayesian analysis, information on the choice of priors and Markov chain Monte Carlo settings
☒	☐	For hierarchical and complex designs, identification of the appropriate level for tests and full reporting of outcomes
☐	☒	Estimates of effect sizes (e.g. Cohen's d, Pearson's r), indicating how they were calculated

Our web collection on statistics for biologists contains articles on many of the points above.

Software and code

Policy information about availability of computer code

Data collection	TCGA database was used to collect patient-related data.

Data analysis	The protein interaction and complex data were access directly from the:<br>STRING Version11.5 (https://cn.string-db.org/),<br>CORUM Version3.0 (https://mips.helmholtz-muenchen.de/corum/#),<br>UniProt (https://www.uniprot.org/).<br>The final confirmed PPI network was assessed by the STRING database, and the recognized individuals were interacted by Cytoscape Version 3.9.1 software.<br>The raw MS files were analyzed and searched against protein database based on the species of the samples using MaxQuant (1.6.2.10).<br><br>AML data were used to perform validation with the database (http://gibk21.bse.kyutech.ac.jp/PrognoScan/index.html) and Gene Expression Profiling Interactive Analysis (GEPIA) database (http://gepia.cancer-pku.cn).<br><br>We stitched raw enhancer together with aligned H3K27ac and input reads were used to run the ROSE algorithm. Then classified the genes as SE-associated genes by Rose GeneMapper to annotate the genes within the 50-kb range of the super-enhancers.<br><br>Fastq files were trimmed adaptors by TrimGalore and aligned to mm9 reference genome using Bowtie2 with default parameters. Reads with a map score less than 30 and PCR duplications were filtered out by using Samtools. Reads aligned to the regions in ENCODE blacklist were discarded through bedtools. Peaks were called with macs240 (parameters: '-g mm -q 0.05 -m 5 50') using input as control. DiffBind was used to analyze differential binding sites.

For manuscripts utilizing custom algorithms or software that are central to the research but not yet described in published literature, software must be made available to editors and reviewers. We strongly encourage code deposition in a community repository (e.g. GitHub). See the Nature Portfolio guidelines for submitting code & software for further information.

Data

Policy information about availability of data

All manuscripts must include a data availability statement. This statement should provide the following information, where applicable:

- Accession codes, unique identifiers, or web links for publicly available datasets
- A description of any restrictions on data availability
- For clinical datasets or third party data, please ensure that the statement adheres to our policy

The datasets presented in this study can be found in online repositories. The names of the repository/repositories and accession number can be found below: NCBI BioProject PRJNA876046. All other relevant data are available within the article file, Supplementary Figures or Supplementary data file, or available from the authors on reasonable request. Uncropped scans of the bolts were shown in Supplementary Fig. 7.

Human research participants

Policy information about studies involving human research participants and Sex and Gender in Research.

Reporting on sex and gender	The study did not involve human research participants.
Population characteristics	-
Recruitment	-
Ethics oversight	-

Note that full information on the approval of the study protocol must also be provided in the manuscript.

Field-specific reporting

Please select the one below that is the best fit for your research. If you are not sure, read the appropriate sections before making your selection.

[x] Life sciences   [ ] Behavioural & social sciences   [ ] Ecological, evolutionary & environmental sciences

For a reference copy of the document with all sections, see nature.com/documents/nr-reporting-summary-flat.pdf

Life sciences study design

All studies must disclose on these points even when the disclosure is negative.

Sample size	No statistical method was used to determine the sample size. Sample size was chosen based on standards in the field. Samples size and number of independent experiments are stated in figure legends or in Methods section.
Data exclusions	No data were excluded from analyses.
Replication	ChIP-seq and IP-MS has two replication, RT-qPCR has three replication.

Randomization	Sample/animal allocation were randomized.
Blinding	Where possible blinded analysis of data was performed. Due to different phenotype and timepoint, collection of mouse model was not blinded, but since blinded data approaches were used, where applicable we believe this has no effect on data quality.

Reporting for specific materials, systems and methods

We require information from authors about some types of materials, experimental systems and methods used in many studies. Here, indicate whether each material, system or method listed is relevant to your study. If you are not sure if a list item applies to your research, read the appropriate section before selecting a response.

Materials & experimental systems

n/a	Involved in the study
☐	☒ Antibodies
☐	☒ Eukaryotic cell lines
☒	☐ Palaeontology and archaeology
☐	☒ Animals and other organisms
☒	☐ Clinical data
☒	☐ Dual use research of concern

Methods

n/a	Involved in the study
☐	☒ ChIP-seq
☐	☒ Flow cytometry
☒	☐ MRI-based neuroimaging

Antibodies

Antibodies used	CAPG antibody (ab181092, abcam) were used in ChIP-seq and IP-MS.<br>Normal Rabbit IgG (millipore, 12-370) were used in IP-MS.<br>MLL1(Active Motif,61296), DBC1(ZenBio, R24080), ZFP91(ZenBio, 252448), MCRS2(ZenBio, 127292), EEF1G(ZenBio, 389310), RPL4 (Abclonal, A5886), WDR5(Active Motif,61486), GAPDH(CST,2118), HRP Goat Anti-Rabbit IgG (H+L)(Abclonal, AS014) were used in western blot.<br>Monoclonal antibodies to Mac-1 (M1/70, Biolegend), Gr-1 (RB6-8C5, Biolegend), c-Kit (2B8, Biolegend), Lin mix (Gr1, CD4, CD3, CD8a, Ter119, B220, IgM) (Biolegend), CD34 (MEC14.7, Biolegend), Sca1 (D7, Biolegend), FcgRII/III (93, Biolegend), IL-7Ra (A7R34, Biolegend) (all used as 50 ng per million cells) were used where indicated
Validation	More detailed information about these antibodies ais available on these manufacturers' websites.

Eukaryotic cell lines

Policy information about cell lines and Sex and Gender in Research

Cell line source(s)	THP-1
Authentication	Commercial cell lines-THP-1: ECACC listed and purchased from Solarbio(SCC-121812), cell line were provided with CoA/COO upon shipping .
Mycoplasma contamination	Cell lines tested negative for mycoplasma contamination.
Commonly misidentified lines (See ICLAC register)	-

Animals and other research organisms

Policy information about studies involving animals; ARRIVE guidelines recommended for reporting animal research, and Sex and Gender in Research

Laboratory animals	C57BL/6 14weeks
Wild animals	-
Reporting on sex	There was no gender bias in this study.
Field-collected samples	The study did not involve samples collected from the field.
Ethics oversight	The ethics committee of the Peking university fist hospital.

Note that full information on the approval of the study protocol must also be provided in the manuscript.

ChIP-seq

Data deposition

- [x] Confirm that both raw and final processed data have been deposited in a public database such as GEO.
- [x] Confirm that you have deposited or provided access to graph files (e.g. BED files) for the called peaks.

Data access links<br>*May remain private before publication.*	NCBI BioProject PRJNA876046.
Files in database submission	The datasets presented in this study can be found in online repositories. The names of the repository/repositories and accession number(s) can be found below: NCBI BioProject PRJNA876046.
Genome browser session<br>(e.g. UCSC)	NCBI BioProject PRJNA876046

Methodology

Replicates	Two replicates for each experiment.
Sequencing depth	6G every replication.
Antibodies	Abcam, ab181092
Peak calling parameters	Fastq files were trimmed adaptors by TrimGalore and aligned to mm9 reference genome using Bowtie2 with default parameters. Reads with a map score less than 30 and PCR duplications were filtered out by using Samtools. Reads aligned to the regions in ENCODE blacklist were discarded through bedtools. Peaks were called with macs240 (parameters: '-g mm -q 0.05 -m 5 50') using input as control. DiffBind was used to analyze differential binding sites.
Data quality	Fastq files were trimmed adaptors by TrimGalore and aligned to mm9 reference genome using Bowtie2 with default parameters. Reads with a map score less than 30 and PCR duplications were filtered out by using Samtools. Reads aligned to the regions in ENCODE blacklist were discarded through bedtools. Peaks were called with macs240 (parameters: '-g mm -q 0.05 -m 5 50') using input as control. DiffBind was used to analyze differential binding sites.
Software	Fastq files were trimmed adaptors by TrimGalore and aligned to mm9 reference genome using Bowtie2 with default parameters. Reads with a map score less than 30 and PCR duplications were filtered out by using Samtools. Reads aligned to the regions in ENCODE blacklist were discarded through bedtools. Peaks were called with macs240 (parameters: '-g mm -q 0.05 -m 5 50') using input as control. DiffBind was used to analyze differential binding sites.

Flow Cytometry

Plots

Confirm that:

- [x] The axis labels state the marker and fluorochrome used (e.g. CD4-FITC).
- [x] The axis scales are clearly visible. Include numbers along axes only for bottom left plot of group (a 'group' is an analysis of identical markers).
- [x] All plots are contour plots with outliers or pseudocolor plots.
- [x] A numerical value for number of cells or percentage (with statistics) is provided.

Methodology

Sample preparation	Take 20–30 μL of peripheral blood through the tail vein of the mouse and add to the anticoagulation tube. Take the bone marrow cells from the femur and tibia of the sacrificed mice. The red blood cells were lysed, and the bone marrow cells were filtered using a 100-mm cell strainer.
Instrument	Attune NxT flow cytometer
Software	The results were analyzed using FlowJo software.
Cell population abundance	At least 10^5 cells per experiment, all antibodies used 50 ng per million cells.
Gating strategy	Wild-type cells from bone marrow were used as a negative control.

- [x] Tick this box to confirm that a figure exemplifying the gating strategy is provided in the Supplementary Information.
